# TiO_2_ Coated ZnO Nanorods by Mist Chemical Vapor Deposition for Application as Photoanodes for Dye-Sensitized Solar Cells

**DOI:** 10.3390/nano9091339

**Published:** 2019-09-19

**Authors:** Qiang Zhang, Chaoyang Li

**Affiliations:** 1 School of Systems Engineering, Kochi University of Technology, Kami, Kochi 782-8502, Japan; 216005z@gs.kochi-tech.ac.jp; 2 Center for Nanotechnology, Kochi University of Technology, Kami, Kochi 782-8502, Japan

**Keywords:** zinc oxide, titanium dioxide, core–shell nanorods, chemical bath deposition, mist chemical vapor deposition

## Abstract

In this study, a mist chemical vapor deposition method was applied to create a coating of titanium dioxide particles in order to fabricate ZnO/TiO_2_ core–shell nanostructures. The thin layers of titanium dioxide on the zinc oxide nanorods were uniform and confirmed as pure anatase phase. The morphological, structural, optical and photoluminescence properties of the ZnO/TiO_2_ core–shell structures were influenced by coating time. For instance, the crystallinity of the titanium dioxide increased in accordance with an increase in the duration of the coating time. Additionally, the thickness of the titanium dioxide layer gradually increased with the coating time, resulting in an increased surface area. The transmittance of the arrayed ZnO/TiO_2_ core–shell structures was 65% after 15 min of coating. The obtained ZnO/TiO_2_ core–shell nanostructures demonstrated high potentiality to serve as photoanodes for application in dye-sensitized solar cells.

## 1. Introduction

Since Brian O’Regan and Michael Grätzel developed dye-sensitized solar cells (DSSCs) in 1991 [1], DSSCs have been widely investigated as a promising alternative to conventional photovoltaic devices due to their low fabrication cost, nontoxicity and promising conversion efficiency [2,3]. Hitherto, titanium dioxide (TiO_2_)-based DSSCs have been reported to achieve a notable power conversion efficiency (PCE) of more than 14% [4]. In order to improve the PCE, four dominant functions—specifically the photoanodes, dye, electrolyte and counter electrodes—need to be optimized. Photoanodes in particular have a strong influence on the PCE of DSSCs due to their electron injection and transportation processes. Further improvement in the PCE of TiO_2_-based DSSCs is difficult due to the intrinsic properties of TiO_2_, such as the difficulty of constructing TiO_2_ nanostructures, which limits the surface area of TiO_2_ photoanodes in a DSSC. The low carrier transportation rate of TiO_2_ also leads to low electron mobility in photoanodes [5,6]. Recently, zinc oxide (ZnO) has attracted much attention as an alternative photoanode material because it exhibits a similar bandgap (3.37 eV at room temperature) and electron injection process from excited dyes as TiO_2_ [7]. Additionally, ZnO achieves a much higher electron mobility (200~1000 cm^2^/(V∙s)) than TiO_2_ (0.1~4 cm^2^/(V∙s)) [8], which may enhance its electron transportation. Moreover, ZnO is much easier to fabricate into various nanostructures with good alignment, which increases its light absorption [9]. Therefore, ZnO is expected to be a promising photoanode material to improve the conversion efficiency of DSSCs. Compared with random ZnO particles, one-dimensional ZnO nanostructures, including nanotubes, nanorods and nanowires, are intended to facilitate fast electron transfer and thereby reduce electron recombination in DSSCs [10].

It has been reported that the highest power conversion efficiency of a pure ZnO-based DSSC is 7.5% [10,11], which is much lower than that of a TiO_2_-based DSSC [4]. First, due to its poor chemical stability, ZnO can be easily dissolved in both acidic and strong alkaline solutions at room temperature. Therefore, pure ZnO photoanodes are easy to dissolve in acidic dye and electrolyte solutions. Moreover, some defects of ZnO increase electron–hole recombination and Zn^2+^/dye complex formation at the interface [12,13,14,15]. One solution to overcome such disadvantages is to fabricate a core–shell structure by coating a chemically stable shell onto ZnO. This core–shell structure forms an energy barrier that can reduce electron—hole recombination and passivate the ZnO surface, thereby reducing the formation of the Zn^2+^/dye complex [16]. Compared with ZnO, TiO_2_ shows excellent chemical stability; it is stable in all alkaline solutions and most acidic solutions (except hydrofluoric acid) at room temperature. By coating a TiO_2_ shell onto the whole surface of a ZnO photoanode, the chemical stability of the photoanode should be significantly improved (equal to the chemical stability of TiO_2_), while the electron–hole recombination on the surface of the ZnO photoanode should also be reduced. Therefore, ZnO/TiO_2_ core–shell nanorods are one of the most promising nanocomposites. It has been reported that the PCE of DSSCs with pure ZnO photoanodes can be improved by about 1 to 5 times by coating a TiO_2_ shell onto the ZnO photoanodes [17,18,19,20]. Among the three phases of TiO_2_, brookite phase TiO_2_ is the most difficult to fabricate into a thin film [21,22], while anatase phase TiO_2_ has a band gap (~3.2 eV) more similar to ZnO (~3.37 eV) than rutile phase TiO_2_ (~3.0 eV) [23,24]. Previous studies have reported that ZnO nanorods can be coated with a TiO_2_ layer by the solution method, the sol-gel method or atomic layer deposition [25,26,27]. However, control of the phase, thickness and uniformity of the TiO_2_ layer remain unsolved issues. According to our previous research [28,29], mist chemical vapor deposition (mist CVD) [30] is an effective method of synthesizing pure anatase phase TiO_2_ thin films.

In this study, the mist CVD system consisted of two stages: A stage involving the creation of mist droplets and a deposition stage (in a reaction chamber). In the first stage, mist droplets were transformed from a solution of precursors by three ultrasonic transducers (2.4 MHz). Then, the mist droplets were transported into a reaction chamber by carrier and dilution gases. During transportation in the specially-designed reaction chamber, the size of the mist droplets was decreased from the microscale to the nanoscale. The nanoscale was maintained until deposition. Due to gravity and adsorption, the mist droplets moved effectively onto the substrate, which was set in the reaction chamber with the appropriate heating [28,30].

In our previous research, well-arrayed ZnO nanorods with high transmittance (over 70% in the visible region) were fabricated using a chemical bath deposition (CBD) method [31,32,33]. Here, in order to obtain photoanodes with excellent chemical stability, high transmittance, and high electron mobility, we employed both the mist CVD and CBD methods to fabricate ZnO/TiO_2_ core–shell nanorods by coating ZnO nanorods with TiO_2_ particles.

## 2. Materials and Methods

### 2.1. Fabrication of ZnO/TiO_2_ Core–Shell Nanorods

Aluminum oxide (2 wt.%)-doped ZnO (AZO) thin films with 300 nm thickness were deposited on alkali-free glass sheets (Eagle XG, Corning Inc., Corning, NY, USA) by a conventional 13.56 MHz radio frequency (RF) magnetron sputtering system. Following the deposition of the AZO films, ZnO nanorods were fabricated on the AZO substrates by CBD. During the CBD process, the substrates were immersed in a solution containing Zn(NO_3_)_2_ and hexamethylenetetramine (C_6_H_12_N_4_, HMTA) for 5 h at 95 °C. The deposition conditions for the AZO film and ZnO nanorods are shown in Table 1 and Table 2, respectively. After deposition, the obtained ZnO nanorods were coated with TiO_2_ by the mist CVD method. The deposition conditions for coating are shown in Table 3.

### 2.2. Characterization

The morphological properties of the ZnO nanorods were evaluated at 5 kV by a field emission scanning electron microscope (FESEM, SU-8020, Hitachi, Tokyo, Japan). Energy dispersive X-ray spectroscopy (EDS) analysis was performed at 15 kV using a silicon drift detector (SDD, X-Max, Horiba, Tokyo, Japan) fitted to the FESEM. The structural properties were investigated by grazing incidence X-ray diffraction (GIXRD, ATX-G, Rigaku, Tokyo, Japan) with an incidence angle of 0.35° and Raman spectroscopy (LabRAM HR-800, Horiba Jobin Yvon, Longjumeau, France) with a 532.8 nm excitation laser. Transmittance measurements were acquired using a spectrophotometer (U-4100, Hitachi, Tokyo, Japan). The photoluminescence (PL) spectra were acquired by a micro-PL/Raman spectroscope (iHR320, Horiba, Tokyo, Japan) with a 325 nm He-Cd laser as the excitation light source. All measurements were carried out at room temperature.

## 3. Results

**Figure 1:**
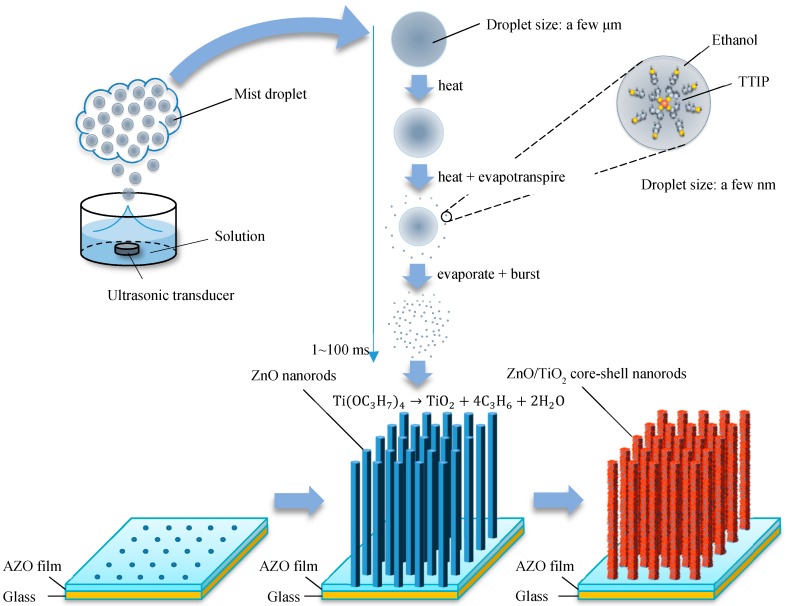
Mechanism of ZnO/TiO_2_ core–shell nanorod fabrication.

The proposed mechanism of ZnO/TiO_2_ core–shell nanorod fabrication is shown in Figure 1. First, the ZnO nanorods were prepared on the AZO film, which acted as a seed layer, by the CBD method. After the CBD process, the obtained ZnO nanorods were placed in the reaction chamber for mist CVD. During the mist CVD process, the mist droplets—including both titanium tetraisopropoxide (TTIP) and ethanol—were transformed from the solution of precursors by ultrasonic transducers. Following this, the mist droplets were transported onto the surface of the ZnO nanorods by carrier and dilution gases. During transportation of the mist droplets in the reaction chamber, the size of the mist droplets decreased from a few micrometers to a few nanometers under the influence of heat, evapotranspiration and burst. Finally, anatase phase TiO_2_ particle shells were deposited on the surface of the ZnO nanorods due to the pyrolysis reaction of TTIP at 400 °C [28].

**Figure 2:**
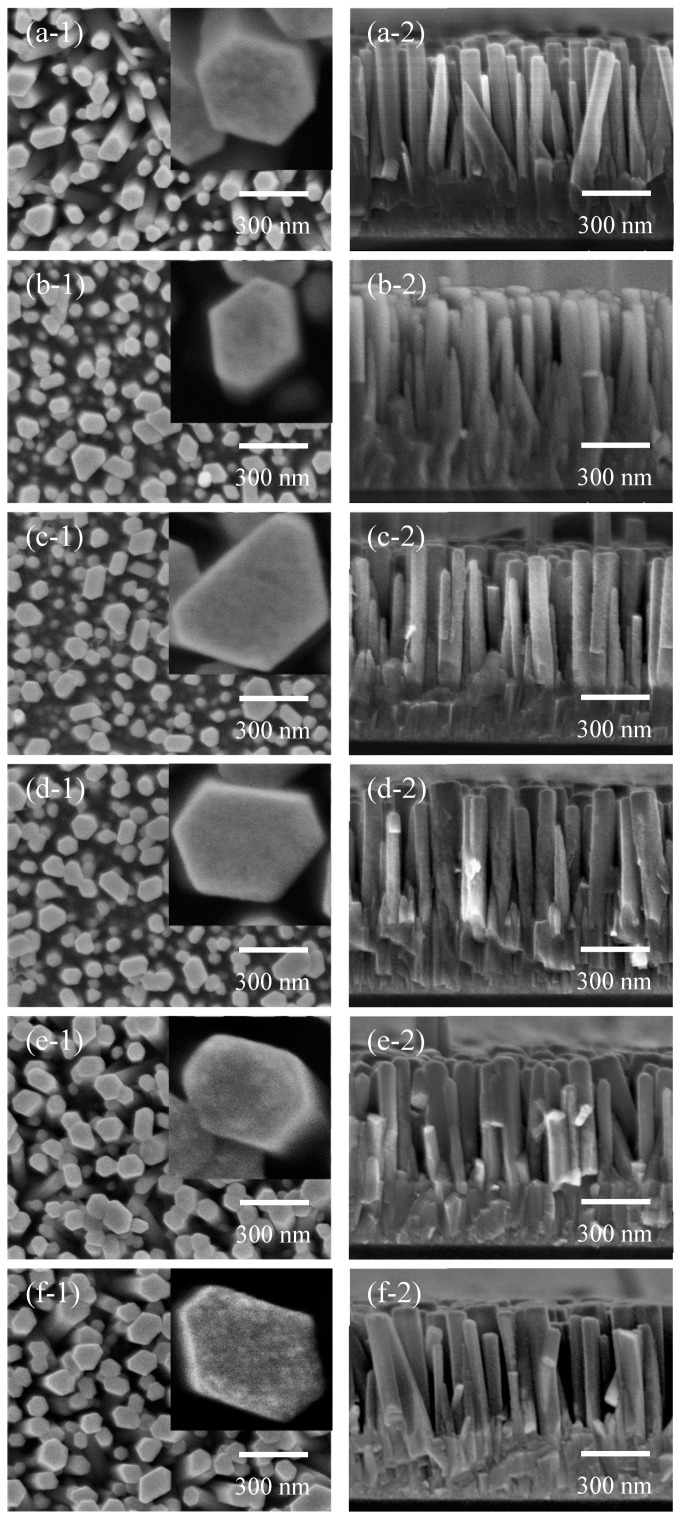
SEM images of the as-deposited ZnO nanorods (**a**) and ZnO nanorods coated with TiO_2_ by mist chemical vapor deposition (CVD) for (**b**) 30 s; (**c**) 2 min; (**d**) 5 min; (**e**) 10 min; and (**f**) 15 min ((**1**) top view and (**2**) cross section view).

Figure 2 shows SEM images of the as-deposited ZnO nanorods and ZnO nanorods coated with TiO_2_ by mist CVD. From the top view images, uniform ZnO nanorods with hexagonal structure were obtained on the AZO substrate after the CBD process. The inset images in the top view images show the surface of a single ZnO nanorod. Compared with the as-deposited ZnO nanorods, no obvious changes were observed on the surface of the nanorods coated with TiO_2_ for 30 s, 2 min and 5 min. When the TiO_2_ coating time was increased to 10 min and 15 min, TiO_2_ particles were observed on the surface of the ZnO nanorods. Compared with the relatively smooth surface of the as-deposited ZnO nanorods, the ZnO nanorods coated with TiO_2_ for 15 min showed a rough surface with many TiO_2_ particles, indicating the surface area of the nanorods had increased. A larger surface area of photoanodes in a DSSC enhances its adsorption of dye, which may improve the PCE of the DSSC. Based on the cross section view images, it appeared the ZnO nanorods grew vertically on the AZO substrates, and the average length of the ZnO nanorods was around 600 nm. The SEM results revealed that the TiO_2_ particle layers on the ZnO nanorods were uniform.

**Figure 3:**
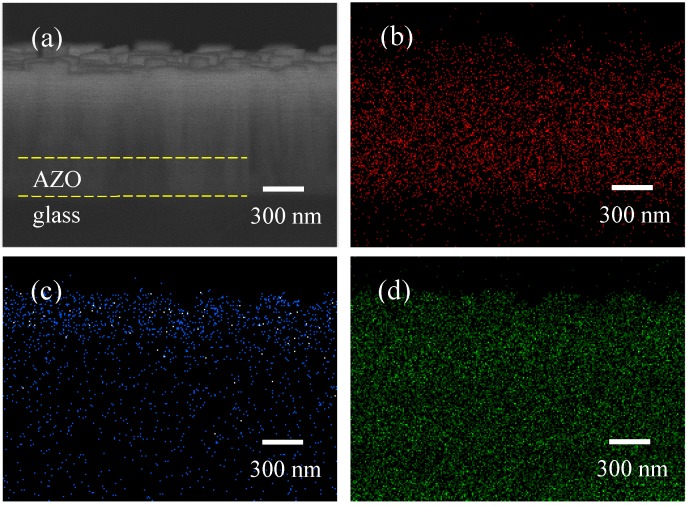
The energy dispersive X-ray spectroscopy (EDS) elemental mapping images of the ZnO nanorods coated with TiO_2_ for 10 min ((**a**) a field emission scanning electron microscope (FESEM) image; (**b**) a zinc element mapping image; (**c**) a titanium element mapping image; and (**d**) an oxygen element mapping image).

EDS elemental mapping images of the ZnO nanorods coated with TiO_2_ for 10 min are shown in Figure 3. The elemental mappings of zinc, titanium and oxygen are shown in Figure 3b–d, respectively. As shown in Figure 3c, elemental titanium was confirmed from the bottom to the top of the ZnO nanorods, indicating that the whole surface of the nanorods was successfully coated with TiO_2_ particles. Considering electron mobility and chemical stability are essential attributes of materials, the TiO_2_ shell that covers the whole surface of a ZnO nanorod will significantly improve the chemical stability of ZnO photoanodes, and the ZnO nanorod inside the TiO_2_ shell will significantly improve the electron mobility of TiO_2_ photoanodes. This TiO_2_ shell is also capable of reducing electron–hole recombination on the surface of ZnO photoanodes. Therefore, the fabrication of ZnO/TiO_2_ core–shell nanorods solves the shortcomings of TiO_2_ photoanodes (low electron mobility) and ZnO photoanodes (poor chemical stability).

**Figure 4:**
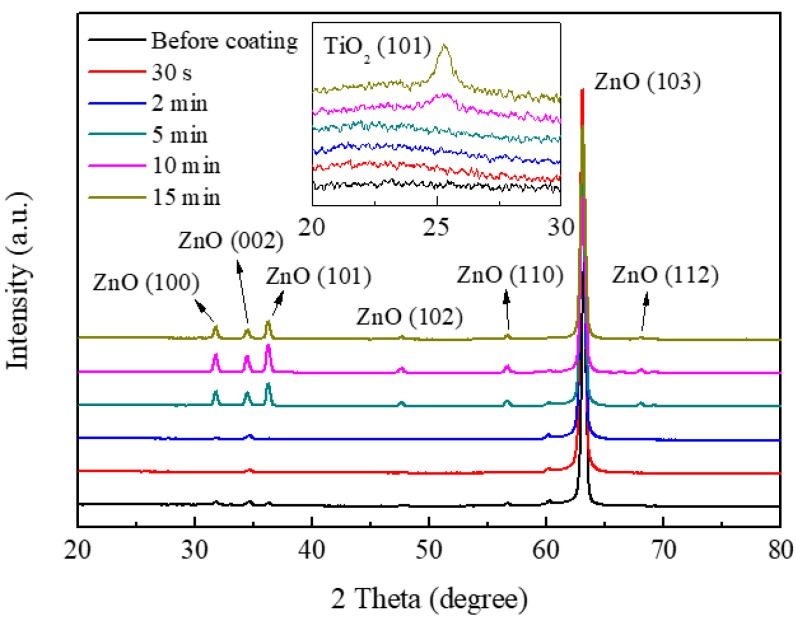
Grazing incidence X-ray diffraction (GIXRD) patterns of the as-deposited ZnO nanorods and ZnO nanorods coated with TiO_2_ by mist CVD.

Figure 4 shows the GIXRD patterns of as-deposited ZnO nanorods and ZnO nanorods coated with TiO_2_ by mist CVD. The observed peaks corresponded with the diffractions from (100), (002), (101), (102), (110), (103), and (112) crystal planes of ZnO. It was clear that the (103) peaks were the dominant peaks, which was due to the low incidence angle (0.35°) employed in the GIXRD analysis. After coating TiO_2_ for 30 s and 2 min, the intensity of the ZnO peaks between 30° and 40° decreased, suggesting the growth of TiO_2_ on the surface of the ZnO nanorods. When the TiO_2_ coating time was increased from 2 min through each time interval to 15 min, an increasing trend in the intensity of the ZnO peaks was observed. This could be attributed to annealing effects during the mist CVD process, which was operated at 400 °C. The crystallinity of the ZnO nanorods was improved by the annealing effects. The inset image in Figure 4 shows the details of the GIXRD patterns from 20° to 30°. In this inset, no peak of TiO_2_ may be observed from the ZnO nanorods coated with TiO_2_ for 30 s, 2 min or 5 min, which may be due to insufficient thickness of the TiO_2_ layer. A (101) diffraction peak, which corresponds with anatase phase TiO_2_, was observed at 2 θ of 25.3° when the TiO_2_ coating time was increased to 10 min and 15 min. The intensity of the TiO_2_ (101) diffraction peak increased as the TiO_2_ coating time increased, indicating the thickness of the TiO_2_ layer increased.

**Figure 5:**
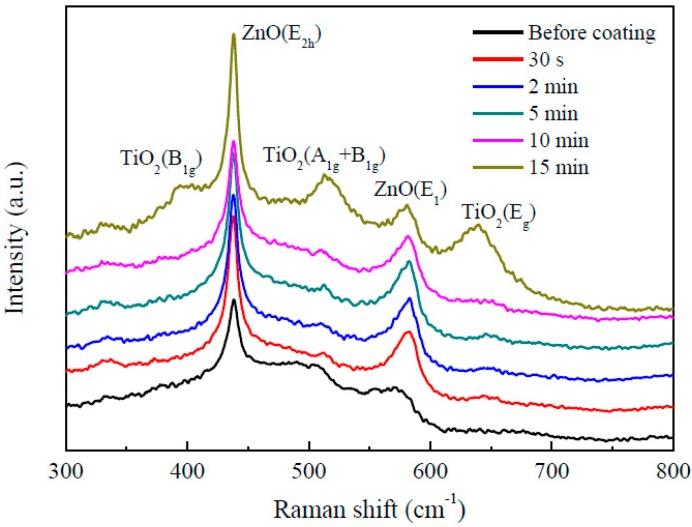
Raman spectra of the as-deposited ZnO nanorods and ZnO nanorods coated with TiO_2_ by mist CVD.

Figure 5 shows the Raman spectra of the as-deposited ZnO nanorods and ZnO nanorods coated with TiO_2_ by mist CVD. The peaks at 438 cm^−1^ and 582 cm^−1^ were assigned to the E_2h_ mode and E_1_ (LO) mode of ZnO, respectively. Three more peaks were observed in the Raman spectrum for the ZnO nanorods coated with TiO_2_ for 15 min. The peaks at 398 cm^−1^, 639 cm^−1^ and 515 cm^−1^ corresponded respectively with the B_1g_ mode, E_g_ mode and a doublet of the A_1g_ and B_1g_ modes of anatase phase TiO_2_, suggesting the ZnO nanorods were coated with TiO_2_ successfully. This result was in agreement with those from the EDS and GIXRD measurements.

**Figure 6:**
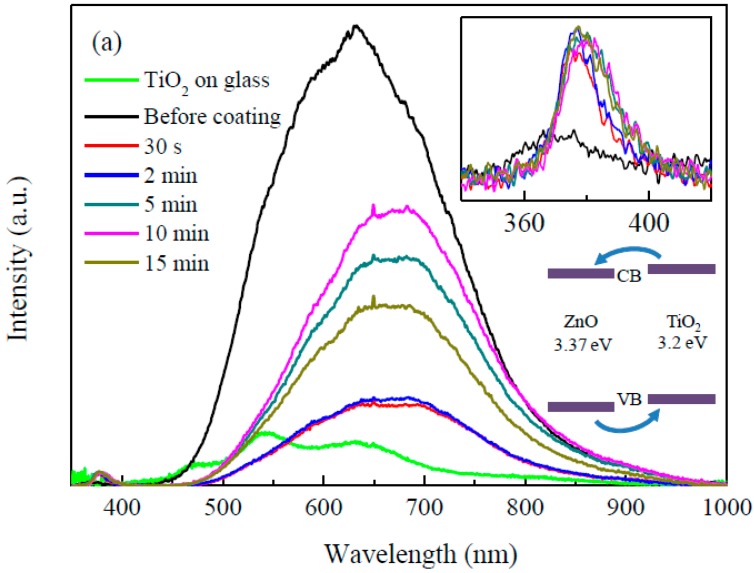

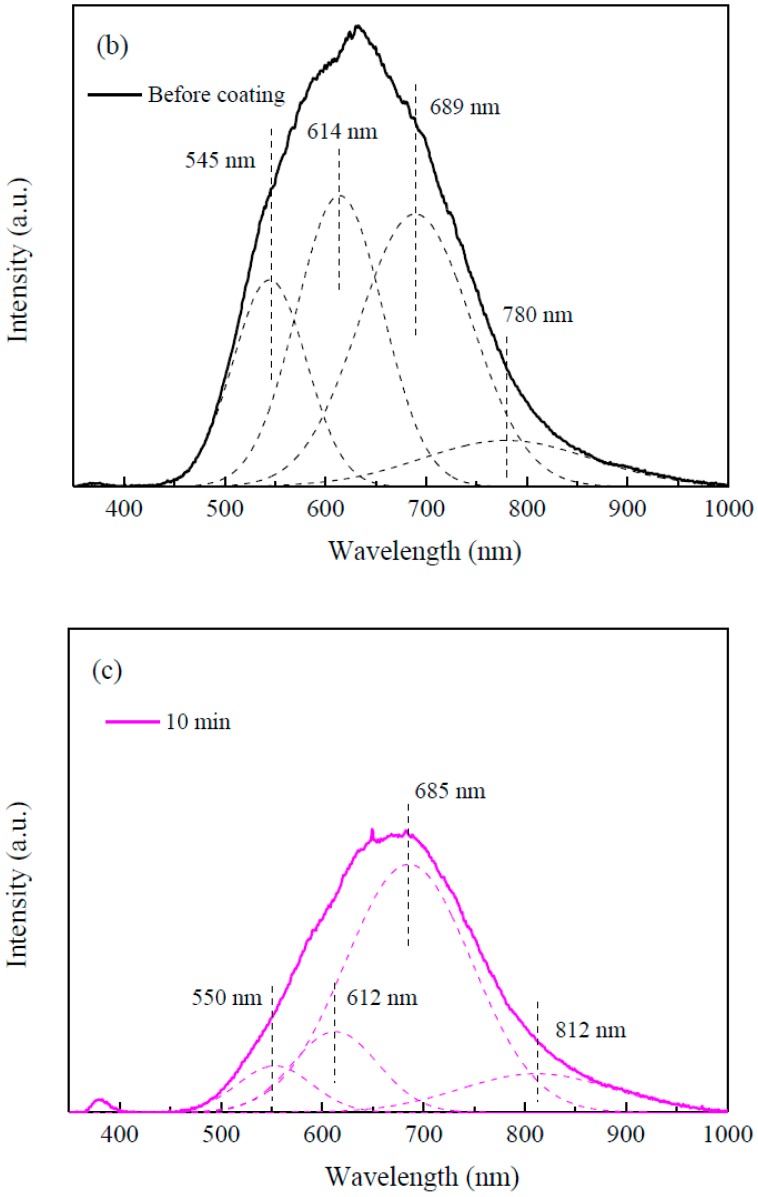
(**a**) Photoluminescence (PL) spectra of the TiO_2_ film on glass, as-deposited ZnO nanorods and ZnO nanorods coated with TiO_2_ by mist CVD; (**b**) PL spectra curve fitting of the as-deposited ZnO nanorods; and (**c**) PL spectra curve fitting of the ZnO nanorods coated with TiO_2_ for 10 min.

PL spectroscopy is a well-established tool for understanding the fate of photoinduced charge carriers and interfacial charge transfer [34,35,36,37]. The PL spectra of the TiO_2_ film on glass, as-deposited ZnO nanorods and ZnO nanorods coated with TiO_2_ by mist CVD are shown in Figure 6a. The inset image shows the details of the PL spectra from 340 nm to 420 nm. The as-deposited ZnO nanorods showed a weak ultraviolet (UV) emission centered at 370 nm and a strong visible emission centered at 632 nm. The UV emission of the as-deposited ZnO nanorods corresponded with the band gap of ZnO (around 3.37 eV). Compared with the as-deposited ZnO nanorods, the UV emission of the ZnO nanorods coated with TiO_2_ for 30 s and 2 min showed a slight red shift. As the TiO_2_ coating time increased from 2 min to 15 min, the UV emission red-shifted gradually to 382 nm, which corresponds with the band gap of anatase phase TiO_2_ (around 3.2 eV). A PL emission of TiO_2_ from 400 nm to 700 nm was not observed for the ZnO nanorods coated with TiO_2_. This PL quenching of TiO_2_ suggests a charge transfer from TiO_2_ to ZnO.

According to the curve fitting for the as-deposited ZnO nanorods visible emission, shown in Figure 6b, the visible emission peak of the as-deposited ZnO nanorods was divided into four emission peaks (dashed line). The emission peaks of the as-deposited ZnO nanorods located at 545 nm (~2.27 eV) and 614 nm (~2.02 eV) may be attributed to oxygen vacancies [38,39,40,41], while the emission peaks located at 689 nm (~1.80 eV) and 780 nm (~1.59 eV) may be attributed to oxygen interstitials [38]. It was observed that the visible emission of the ZnO nanorods showed a red shift after coating with TiO_2_. After the ZnO nanorods were coated with TiO_2_ for 30 s, the peak intensity of the visible emission decreased steeply. As the TiO_2_ coating time increased from 30 s to 15 min, the peak intensity of the visible emission first increased and then decreased. The highest visible emission peak intensity of the ZnO/TiO_2_ core–shell nanorods was obtained with a TiO_2_ coating time of 10 min. The curve fitting of visible emission of the ZnO nanorods coated with TiO_2_ for 10 min is shown in Figure 6c. After curve fitting, the visible emission peak was divided into four emission peaks (dashed line). Similar to the discussion of the as-deposited ZnO nanorods above, the emission peaks located at 550 nm and 612 nm may be attributed to oxygen vacancies in the ZnO nanorods, and the emission peak located at 685 nm may be attributed to oxygen interstitials. The broad emission peak centered at 812 nm (~1.53 eV) may be attributed to oxygen interstitials in the ZnO nanorods and titanium interstitials in the TiO_2_ coating [42,43]. Based on the curve fitting results, the red shift of the ZnO nanorods’ visible emission was attributed to the intensity decrease of the emissions located at around 550 nm and 612 nm, which was due to the annealing effects during the mist CVD process.

The PL results indicated that all of the defects in ZnO could be decreased by coating it with TiO_2_. The oxygen interstitials in ZnO were decreased slightly after coating with TiO_2_. The oxygen vacancies in ZnO were significantly decreased due to the annealing effects during the mist CVD process. Such a decrease in the defects of a photoanode can lead to a decrease in electron–hole recombination, which may, in turn, contribute to a higher PCE of DSSCs.

**Figure 7:**
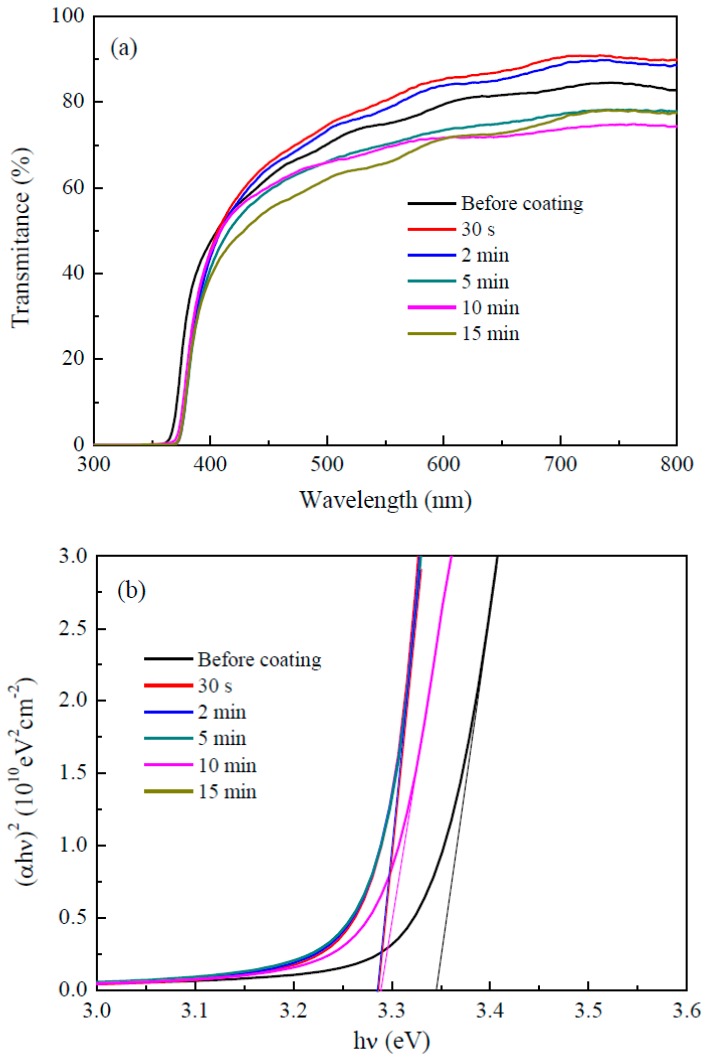
(**a**) Transmission spectra of the as-deposited ZnO nanorods and ZnO nanorods coated with TiO_2_ by mist CVD; and (**b**) variation of (*ahν*)^2^ of the as-deposited ZnO nanorods and ZnO nanorods coated with TiO_2_ as a function of the photon energy (*hν*).

The transmission spectra of the as-deposited ZnO nanorods and ZnO nanorods coated with TiO_2_ by mist CVD are shown in Figure 7a. The transmittance of the as-deposited ZnO nanorods was around 75% in the visible region. The transmittance gradually decreased to around 65% as the TiO_2_ coating time was increased from 30 s to 15 min.

The bandgap (*E_g_*) of the as-deposited ZnO nanorods and ZnO nanorods coated with TiO_2_ could be obtained from the transmittance and absorbance of the ZnO nanorods using the following equations [44]:

(1) $${\left(\alpha h\nu \right)}^{2}=A\left(h\nu -E_{g}\right)$$

(2) $$\alpha =\frac{1}{d}\ln\left(\frac{1}{T}\right)$$

where α is the absorption coefficient, *hν* the incidence photon energy, *A* a constant, *d* the film thickness, and *T* the transmittance. As shown in Figure 7b, a plot of (*αhν*)^2^ as a function of *hν* was made to determine *E_g_* by linear fitting. After fitting, the *E_g_* of the as-deposited ZnO nanorods was around 3.34 eV, which corresponds with the band gap of bulk ZnO (3.37 eV). The *E_g_* of ZnO/TiO_2_ core–shell nanorods was around 3.28 eV, which corresponds with the band gap of anatase phase TiO_2_ (3.2 eV).

## 4. Conclusions

Well-arrayed ZnO/TiO_2_ core–shell nanorods were successfully fabricated on an AZO substrate. We found that mist CVD was a successful method for coating thin layers of TiO_2_ onto ZnO nanorods. The TiO_2_ shells on the ZnO nanorods were confirmed as pure anatase phase, which contributes toward high chemical stability as photoanodes. The surface area of the ZnO nanorods was significantly increased with an increase in the TiO_2_ coating time. The transmittance of the ZnO nanorods decreased from 75% to 65% after 15 min of coating with TiO_2_. The well-arrayed ZnO/TiO_2_ core–shell nanorods contribute to a high transmittance (around 70%), greater electron transfer, greater chemical stability, and a relatively large surface area for dye absorption. As such, these nanorods are expected to be applied as photoanodes to improve the PCE for DSSCs.

## Figures and Tables

**Table 1:** Deposition conditions of aluminum oxide (2 wt.%)-doped ZnO (AZO) film.

Target	AZO (2 wt.%)
Working distance (mm)	60
Working gas, flow rate (sccm)	Argon, 30
Pressure (Pa)	1
Deposition temperature (°C)	150
RF power (W)	60

**Table 2:** Deposition conditions of ZnO nanorods.

Solute	Zn(NO_3_)_2_, HMTA
Solvent	Ultrapure water
Concentration Zn(NO_3_)_2_ (mmol/L)	15
Concentration HMTA (mmol/L)	7.5
Deposition temperature (°C)	95
Deposition time (h)	5

**Table 3:** Deposition conditions of TiO_2_ coating.

Solute	TTIP
Solvent	Ethanol
Concentration (mol/L)	0.10
Deposition temperature (°C)	400
Carrier gas, flow rate (L/min)	Compressed air, 2.5
Dilution gas, flow rate (L/min)	Compressed air, 4.5
Coating time (min)	0.5, 2, 5, 10, 15
